# Association of fibroblast growth factor receptor 1 gene amplification with poor survival in patients with esophageal squamous cell carcinoma

**DOI:** 10.18632/oncotarget.21486

**Published:** 2017-10-04

**Authors:** Dong Wang, Licheng Du, Zhou Wang, Xiangyan Liu, Yejun Qin, Qiangxiu Wang, Zhe Yang, Zhigang Yao, Mo Shi, Bin Shang, Yang Jia, Huaxia Chen, Liang Qiao, Xueqing Wang, Zhaohua Xiao, Zhenchuan Liu

**Affiliations:** ^1^ Department of Thoracic Surgery, Shandong Provincial Hospital Affiliated to Shandong University, Jinan, China; ^2^ Department of General Surgery, Shandong Provincial Hospital Affiliated to Shandong University, Jinan, China; ^3^ Department of Pathology, Shandong Provincial Hospital Affiliated to Shandong University, Jinan, China; ^4^ Department of Oncology, Shandong Provincial Hospital Affiliated to Shandong University, Jinan, China

**Keywords:** esophageal neoplasms, survival analysis, receptor, fibroblast growth factor, type 1

## Abstract

**Purpose:**

To investigate whether *FGFR1* gene amplification is associated with clinicopathologic characteristics and its potential impact on survival in patients with resected esophageal squamous cell carcinoma (ESCC).

**Methods:**

Five hundred fifty-six ESCC patients undergoing curative resection of ESCC were retrospectively studied. *FGFR1* gene copy number was determined in microarrayed tumor samples using fluorescent *in situ* hybridization (FISH) analysis. *FGFR1* gene amplification status was prespecified as copy number ≥ 6 or FGFR1/CEN 8 ratio ≥ 2.2. FGFR1 expression was evaluated by immunohistochemistry. Overall survival (OS) and disease-free survival (DFS) were analyzed using the Kaplan-Meier method followed by the log rank test. Correlation with survival was examined using multivariate Cox regression.

**Results:**

*FGFR1* amplification was identified in 67 (12.1%) patients; these patients had significantly shorter OS (50.0 vs 32.0 months; log rank; *P*<0.001) as well as shorter DFS (47.0 vs 28.0 months; log rank; *P*<0.001) than those without *FGFR1* amplification. Under a Cox proportional hazard model, *FGFR1* amplification was associated with significantly shorter OS (adjusted hazard ratio [AHR]=1.61; 95% CI, 1.10-2.43, *P*=0.004) and DFS (AHR=1.72; 95%CI, 1.15-2.48; *P*<0.001). Moreover, cases with high intratumoral FGFR1 expression showed significantly shorter OS and DFS than those with low FGFR1 expression. The frequency of *FGFR1* amplification was significantly higher in heavy drinkers than in moderate and light drinkers.

**Conclusion:**

*FGFR1* amplification is an independent adverse prognostic factor in surgically resected ESCC. FGFR1 may be a promising therapeutic target in patients with ESCC.

## INTRODUCTION

Esophageal cancer is one of the most aggressive malignancies [1, 2]. Esophageal squamous cell carcinoma (ESCC), the predominant histological type in east Asia, is considered as an environmental malignancy attributable to tobacco smoking and alcohol intake [3]. In contrast, esophageal adenocarcinoma (EAC) is associated with Barrett’s esophagus, and mainly affects Caucasian population [4]. The two main subtypes of esophageal cancer have considerably different epidemiological features but share the same dismal prognosis despite recent advances in imaging and surgical techniques [5].

Over the last decade, molecularly stratified therapy have afforded benefits to patients with many types of cancer but, unfortunately, the same is not true for ESCC [6]. Many genomic abnormalities have been described in ESCC and there is growing evidence of their biological significance [7, 8]. Further delineation of genetic alterations may help uncover aberrant molecular pathways, novel biologic markers and tumorigenic pathways, and eventually allowing successful targeted therapy. Fibroblast growth factor receptor 1 (FGFR1), whose gene is located at 8p12, is a member of the FGFR family of receptor tyrosine kinases (FGFR1-4). FGFRs activation leads to downstream signaling via the PI3K/AKT/RAS/MAPK pathways, which are essential to cell growth, survival, migration, and angiogenesis. *FGFR1* amplification has been identified in breast cancer, head and neck squamous cell carcinoma, ovarian cancer, ESCC, bladder cancer and lung cancer [9–12]. Existing studies on the prognostic impact of *FGFR1* amplification for ESCC have yielded conflicting results. Loga *et al.* investigated the prevalence of *FGFR1* amplification in a tissue microarray containing 346 esophageal adenocarcinomas and 254 ESCCs using dual-labeling fluorescent *in situ* hybridization (FISH) analysis and found that *FGFR1* amplification correlated with the histologic subtype of ESCC (9.4% vs. esophageal adenocarcinoma 1.6%, *P*<0.001) [13]. However, they failed to demonstrate an association between *FGFR1* amplification and clinical outcome. Kwon *et al.* studied 180 patients with resected ESCC and found *FGFR1* amplification in 21.4% (37/173) patients; they observed that *FGFR1* amplification was an independent predictor of prolonged OS in these patients [14]. Kim *et al.* investigated 526 curatively resected ESCC using FISH for *FGFR1* amplification and found that an association between high *FGFR1* amplification (defined as an *FGFR1*/centromer 8 ratio ≥ 2.0, or average number of *FGFR1* signals/tumor cell nucleus ≥ 6.0, or percentage of tumor cells containing ≥ 15 *FGFR1* signals or large cluster in ≥ 10% with significantly shorter disease-free survival (DFS) and OS [15]. FGFR1 inhibition in cell lines and mouse models with *FGFR1*-amplified engrafted tumors suppressed tumor cell growth and induced apoptosis, suggesting that FGFR inhibitors may be an effective therapeutic option in SCCs with *FGFR1* amplification [11, 16, 17]. Furthermore, FGFR1 inhibitors (e.g. dovitinib) have entered early stage clinical trials in patients with solid tumors [18, 19].

In this study, we examined *FGFR1* amplification status and analyzed the impact of *FGFR1* amplification on the OS and DFS in 556 ESCC patients who received radical resection of curative intent at our institution.

## RESULTS

### Demographic and baseline characteristics

**Figure 1:**
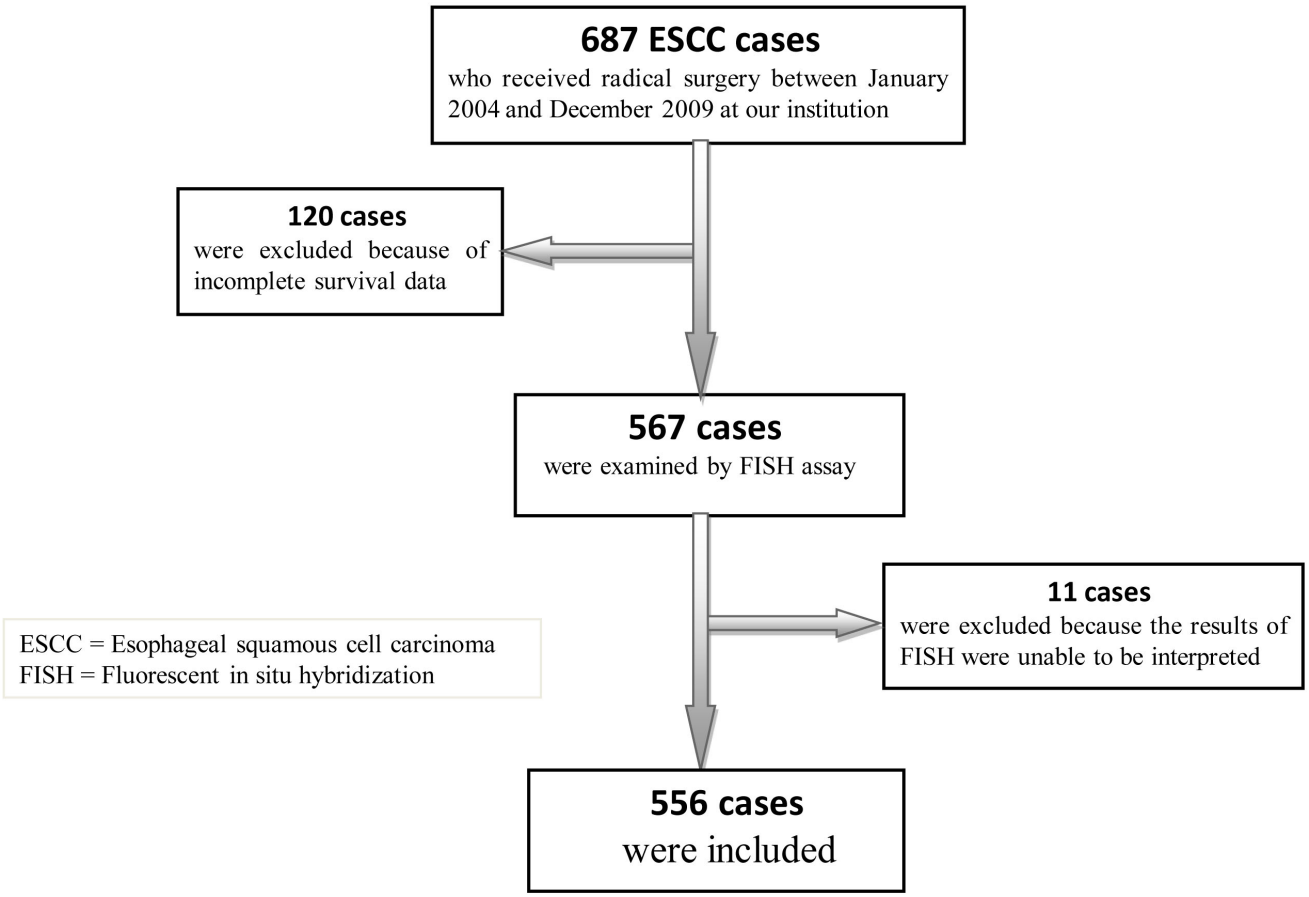
Flowchart of the current study

A total of 687 patients underwent radical resection for ESCC during the study period. 556 cases with undisputable survival data were included in data analysis in the current study (Figure 1). The demographic and baseline characteristics of the study participants are shown in Table 1. The median age of the patients was 63 years (range 39-80 years) and the majority of them (86.2%) were men. The patients had predominantly stage II (58.3%) or III (21.4%) tumors. Slightly more than half of the patients (57.4%) were current smokers and the majority of the patients were moderate (27.3%) or heavy drinkers (48.4%).

**Table 1:** Patient characteristics stratified by *FGFR1* amplification by FISH

Characteristics	All	*FGFR1* amplification		*P*
		No	Yes.	
**No. of patients (%)**	556(100)	489(87.9)	67(12.1)	
**Median age (range), years**	63(39-80)	63(39-80)	64(48-72)	0.45
**Male sex, n(%)**	479(86.2)	418(85.5)	61(91.0)	0.22
**Tumor location, n(%)**				0.43
Upper	9(1.6)	9(1.8)	0(0)	
Middle	429(77.2)	373(76.3)	56(83.6)	
Lower	118(21.2)	107(21.9)	11(16.4)	
**Median tumor size (range), cm**	3.0(0.5-9.0)	3.0(0.5-9.0)	3.5(1.0-8.0)	0.89
**pT stage, n(%)**				0.24
T1	119(21.4)	104(21.3)	15(22.4)	
T2	265(47.7)	238(48.7)	27(40.3)	
T3	141(25.3)	123(25.1)	18(26.9)	
T4	31(5.6)	24(4.9)	7(10.4)	
**pN stage, n(%)**				0.42
N0	210(37.8)	189(38.7)	21(31.3)	
N1	27(48.7)	238(48.6)	33(49.3)	
N2	46(8.3)	38(7.8)	8(11.9)	
N3	29(5.2)	24(4.9)	5(7.5)	
**pTNM stage, n(%)**				0.28
I	113(20.3)	96(19.6)	17(25.4)	
II	324(58.3)	291(59.5)	33(49.2)	
III	119(21.4)	102(20.9)	17(25.4)	
**Differentiation, n(%)**				0.27
Well	177(31.8)	154(31.5)	23(34.3)	
Moderate	286(51.5)	257(52.6)	29(43.4)	
Poorly	93(16.7)	78(15.9)	15(22.4)	
**P53 expression, n(%)**				0.13
Positive	289(52)	260(53.2)	29(43.3)	
**Diabetes, n(%)**				0.92
Yes	77(13.8)	68(13.9)	9(13.4)	
**Hypertension, n(%)**				0.58
Yes	191(34.4)	170(34.8)	21(31.3)	
**Smoking status, n(%)**				0.16
Never-smoker	124(22.3)	115(23.5)	9(13.4)	
Former smoker	113(20.3)	99(20.7)	14(21.0)	
Current smoker	319(57.4)	275(55.8)	44(65.6)	
**Alcohol intake, n(%)**				0.00
None	34(6.1)	34(7.0)	0(0)	
Light	101(18.2)	100(20.5)	1(1.5)	
Moderate	152(27.3)	144(29.4)	8(11.9)	
Heavy	269(48.4)	211(43.1)	58(86.6)	
**Neoadjuvantchemotherapy, n(%)**				0.44
Yes	52(9.4)	44(9.0)	8(11.9)	
**Adjuvant chemotherapy, n(%)**				0.92
Yes	302(54.3)	266(54.4)	36(53.7)	
**Adjuvant radiotherapy, n(%)**				0.78
Yes	31(5.6)	27(5.5)	4(6.0)	

### Treatment

Nine (1.6%) patients with upper thoracic ESCC received three-incision esophagectomy and three-field lymph dissection while the remaining patients (98.4%) received Ivor Lewis esophagectomy and two-field lymph dissection. Three hundred and two patients (54.3%) who had T stage ≥ T3 and/or lymph node metastasis received adjuvant platinum-based chemotherapy. The proportion of patients receiving adjuvant chemotherapy was 14.2% for stage I, 58.0% for stage II, and 82.4% for stage III patients. Thirty-one patients with pathologic evidence of T4 and/or mediastinal lymphatic metastasis received postoperative mediastinal radiotherapy.

### *FGFR1* amplification

Among a total of 556 cases, 67 (12.1%) were positive for *FGFR1* amplification (Supplementary Figure 1). The median *FGFR1* gene copy number in all patients was 3 (range, 2 to 17 copies per cell). The median *FGFR1* gene copy number was 10 (range, 6 to 17) in ESCC patients with *FGFR1* amplification and 2 (range, 2 to 5) in those without *FGFR1* amplification. The median *FGFR1*/CEN8 ratio was 3.6 (range, 2.2 to 8.5) and 1.4 (range, 1.0 to 2.0) for ESCC patients with or without *FGFR1* amplification, respectively. ESCC patients with and without *FGFR1* amplification were comparable in the demographic and baseline variables except that the percentage of heavy drinkers was significantly higher in patients with *FGFR1* amplification than those without *FGFR1* amplification (86.6% vs 43.1%, *P*<0.001) (Table 1).

### Survival and *FGFR1* amplification

**Figure 2:**
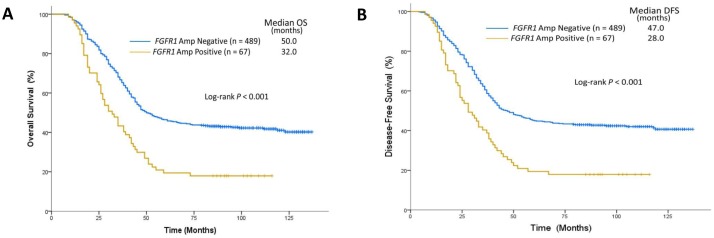
Survival analysis on the basis of FGFR1 amplification status **(A)** Median OS was 32.0 months in the *FGFR1* amplification group (n=67) and 50.0 months in the no amplification group (n=489). **(B)** The median DFS was 28.0 months in the *FGFR1* amplification group (n=67) and 47.0 months in the no amplification group (n=489).

The median follow-up duration for the study cohort was 47.0 months (range, 9.0 to 137.0 months). As expected, both of the median OS and DFS were longer in patients with stage I to II disease than in those with stage III disease (73.0 vs 32.0 months; *P*<0.001; 68.0 vs 28.0 months; *P*<0.001). Kaplan–Meier analysis showed that ESCC patients with *FGFR1* amplification had significantly shorter OS (32.0 months; range, 11.0 to 116.0 months) than those without *FGFR1* amplification (50.0 months; range, 9.0 to 137.0 months) (log rank; *P<*0.001) (Figure 2). After adjustment for sex, pathologic stage, diabetes, adjuvant chemotherapy and other factors, *FGFR1* amplification remained associated with significantly shorter OS (adjusted hazard ratio [AHR], 1.61; 95% CI, 1.10-2.43, *P*=0.004) (Table 2). The median DFS was 42.5 months (range, 5.0 to 137.0 months) for the entire study sample. The median DFS was 28.0 months (range, 7.0 to 116.0 months) for ESCC patients with *FGFR1* amplification and 47.0 (range, 5.0 to 137.0 months) months in those without *FGFR1* amplification. Kaplan-Meier analysis revealed that patients with *FGFR1* amplification had significantly shorter DFS (log rank; *P<*0.001) (Figure 2). After adjusting for sex, pathologic stage, diabetes, and other variables, *FGFR1* amplification was associated with shorter DFS (AHR=1.72; 95%CI, 1.15-2.48; *P<*0.001) (Table 3).

**Table 2:** Multivariate analysis of overall survival of ESCC patients

Variables	Category	OS		
		HR	95%CI	*P*
**Age**	≥ 63 vs.< 63	1.15	0.84 - 1.57	.554
**Sex**	Female vs. male	0.93	0.65 - 1.27	.642
**Pathologic stage**	III vs. I + II	2.83	2.23 - 3.59	.000
**Differentiation**	Poor vs. well/moderate	1.24	0.83 - 1.84	.093
**Diabetes**	Yes vs. no	1.58	0.82 - 2.85	.031
**Hypertension**	Yes vs. no	1.08	0.81 - 1.43	.401
**Adjuvant chemotherapy**	Yes vs. no	0.95	0.77 - 1.19	.679
**Adjuvant radiotherapy**	Yes vs. no	1.16	0.87 - 1.51	.209
**Smoking status**	Smoker vs.never-smoker	1.04	0.82 - 1.29	.747
**Alcohol intake**	Heavy vs. moderate/light	1.17	0.93 - 1.41	.752
***FGFR1* amplification by FISH**	Yes vs. no	1.61	1.10 - 2.43	.004

OS, overall survival; FGFR1, fibroblast growth factor receptor 1; FISH, fluorescent *in situ* hybridization; HR, hazard ratio.

Clinical stage of initial diagnosis was determined according to the American Joint Committee on Cancer (seventh edition) guidelines.

**Table 3:** Multivariate analysis of disease-free survival of ESCC patients

Variables	Category	DFS		
		HR	95%CI	*P*
**Age**	≥ 63 vs.< 63	1.06	0.79 - 1.50	.591
**Sex**	Female vs. male	0.92	0.68 - 1.25	.604
**Pathologic stage**	III vs. I + II	2.82	2.22 - 3.58	.000
**Differentiation**	Poor vs. well/moderate	1.38	0.98 - 1.94	.065
**Diabetes**	Yes vs. no	1.36	0.71 - 2.58	.196
**Hypertension**	Yes vs. no	0.96	0.79 - 1.16	.480
**Adjuvant chemotherapy**	Yes vs. no	0.95	0.76 - 1.19	.673
**Adjuvant radiotherapy**	Yes vs. no	1.19	0.91 - 1.55	.202
**Smoking status**	Smoker vs.never-smoker	1.03	0.83 - 1.29	.777
**Alcohol intake**	Heavy vs. moderate/light	1.14	0.91 - 1.40	.751
***FGFR1* amplification by FISH**	Yes vs. no	1.72	1.15 - 2.48	.000

DFS, disease-free survival; FGFR1, fibroblast growth factor receptor 1; FISH, fluorescent *in situ* hybridization; HR, hazard ratio.

Clinical stage of initial diagnosis was determined according to the American Joint Committee on Cancer (seventh edition) guidelines.

### FGFR1 expression by IHC

**Figure 3:**
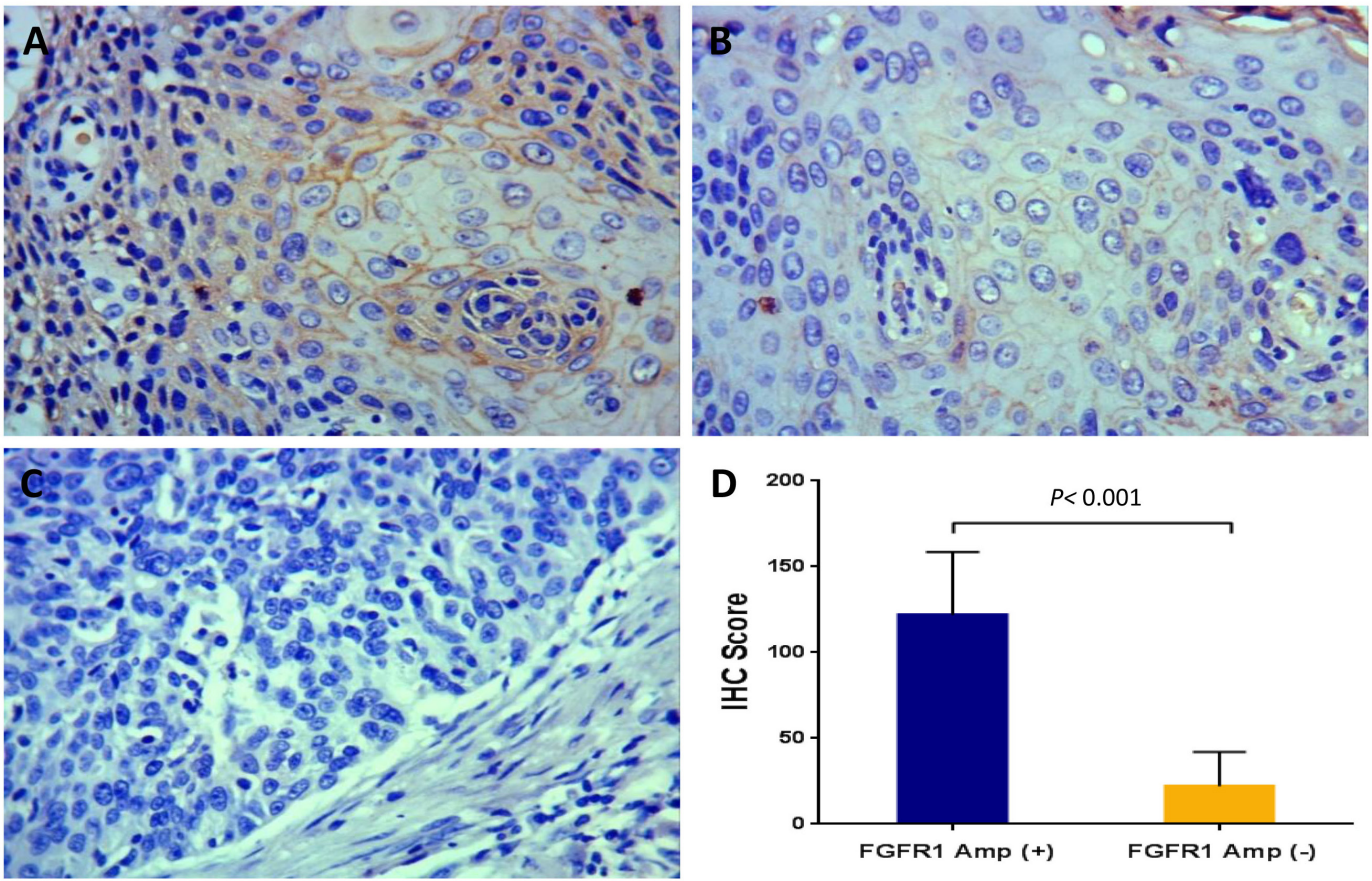
Fibroblast growth factor receptor 1 (FGFR1) expression in esophageal squamous cell carcinomas assessed by immunohistochemistry (magnification: ×200) **(A)** Strong expression. **(B)** Weak expression. **(C)** No expression. **(D)** The correlation between fibroblast growth factor receptor 1 (*FGFR1*) amplification and protein expression: the group with *FGFR1* amplification had a higher expression level than those without *FGFR1* amplification.

**Figure 4:**
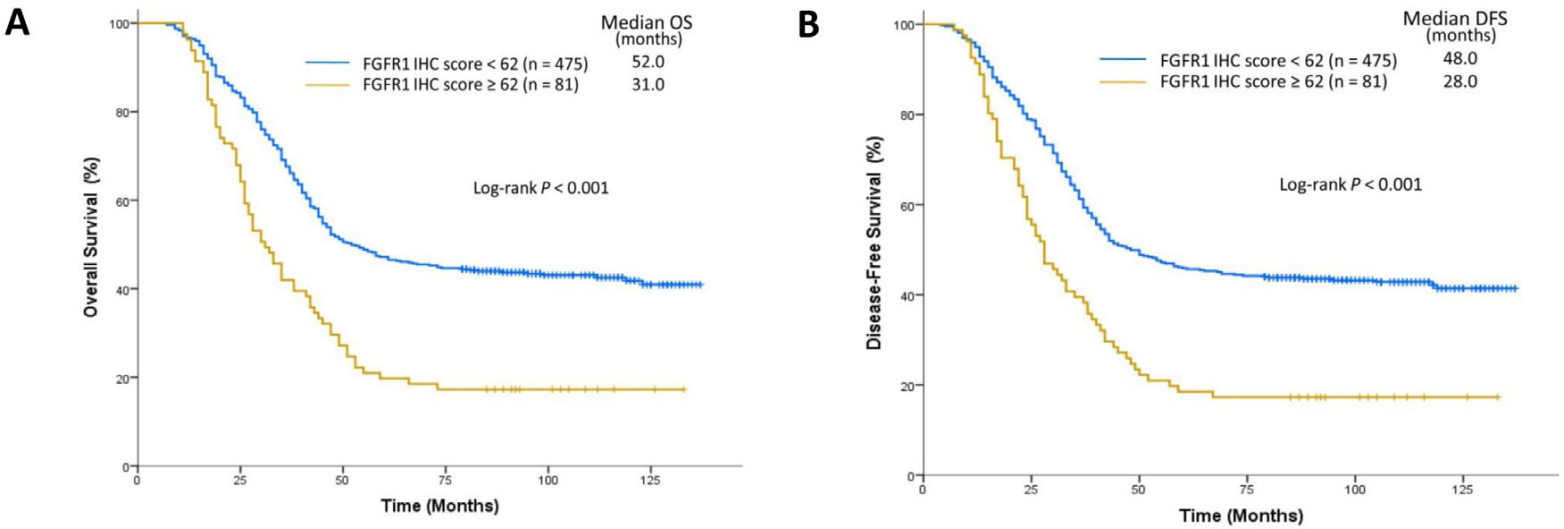
Survival analysis on the basis of FGFR1 expression **(A)** The median OS was 31.0 months in the high FGFR1 expression group (n=81) and 52.0 months in the low FGFR1 expression group (n=475). **(B)** The median DFS was 28.0 months in the high FGFR1 expression group (n=81) and 48.0 months in the low FGFR1 expression group (n=475).

In addition to gene copy number analysis, we also evaluated the correlation between *FGFR1* amplification and FGFR1 expression by using IHC (Figure 3). The mean IHC score for FGFR1 was 34.1 ± 39.5 (range, 0 to 242) for the entire study sample. A cutoff IHC score of 62 was used to stratify ESCC patients into the high FGFR1 expression group (n=81) and the low FGFR1 expression group (n=475). ESCC patients with high FGFR1 expression and those with low FGFR1 expression were comparable in the demographic and baseline variables except that the percentage of heavy drinkers was significantly higher in patients with high FGFR1 expression than those with low FGFR1 expression (79.0% vs 43.2%, *P*<0.001) (Supplementary Table 1, Supplementary Figure 2). Furthermore, ESCC patients with *FGFR1* amplification had significantly higher IHC scores (mean 121.8 ± 36.8; range, 78 to 242) than those without *FGFR1* amplification (mean 22.1 ± 19.9; range, 0 to 75) (*P*<0.001) (Figure 3) and all patients with *FGFR1* amplification fell into the high FGFR1 expression group. Moreover, patients with high intratumoral FGFR1 expression had shorter OS (31.0 vs 52.0 months in subjects with low FGFR1 expression; *P*<0.001) and DFS (28.0 vs 48.0 months; *P*<0.001) (Figure 4).

### *FGFR1* amplification and patient response to adjuvant chemotherapy

**Figure 5:**
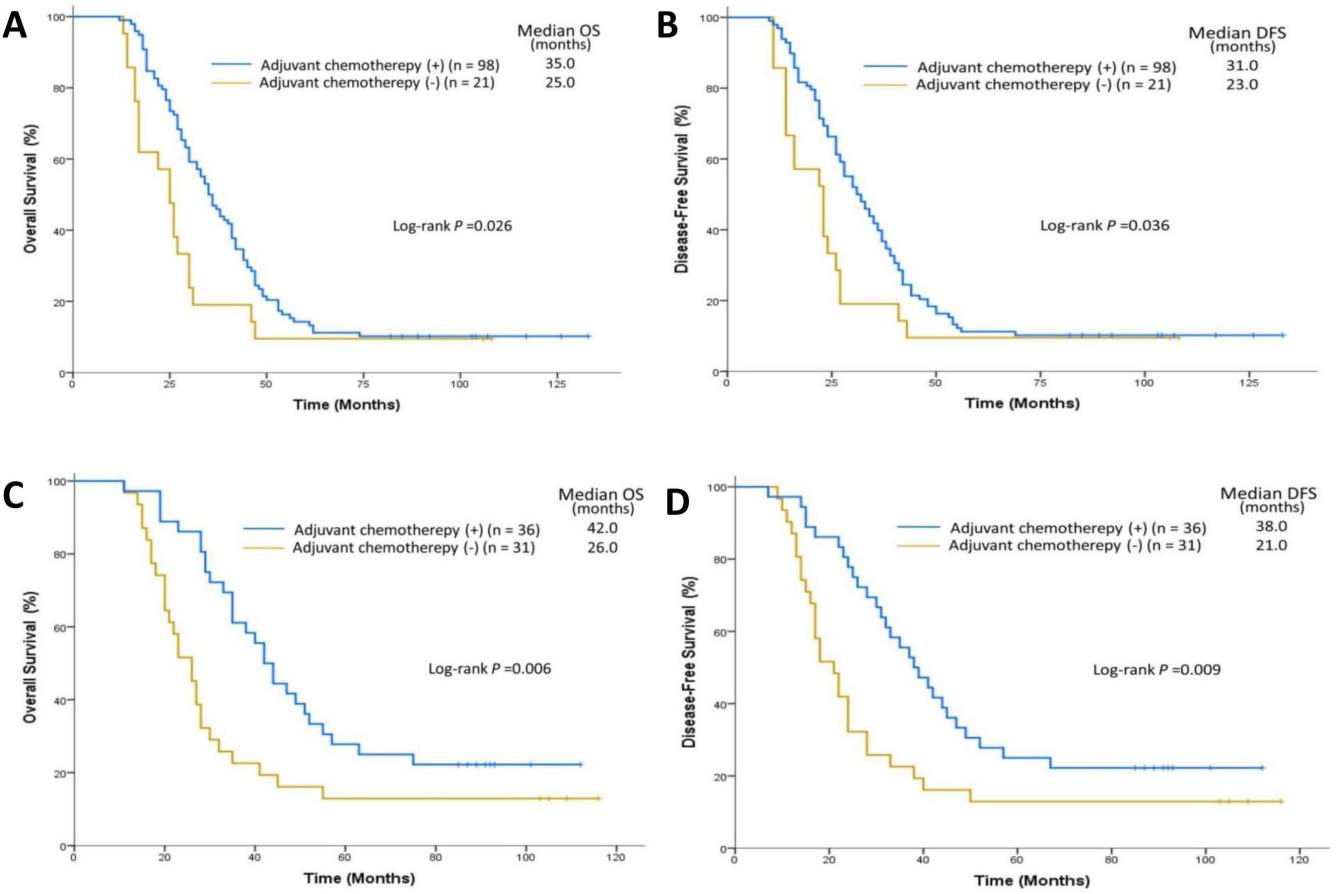
Impact of adjuvant chemotherapy on OS and DFS in patients with stage III ESCC **(A)** The median OS was 35.0 months in the group with adjuvant chemotherapy (n=98) and 25.0 months in the group without adjuvant chemotherapy (n=21). **(B)** The median DFS was 31.0 months in the group with adjuvant chemotherapy (n=98) and 23.0 months in the group without adjuvant chemotherapy (n=21). Impact of adjuvant chemotherapy on OS and DFS in patients with *FGFR1* amplification. **(C)** The median OS was 42.0 months in the group with adjuvant chemotherapy (n=36) and 26.0 months in the group without adjuvant chemotherapy (n=31). **(D)** The median DFS was 38.0 months in the group with adjuvant chemotherapy (n=36) and 21.0 months in the group without adjuvant chemotherapy (n=31).

We assessed the impact of neoadjuvant and adjuvant chemotherapy on postoperative survival according to histologic stage. Among 52 patients who received neoadjuvant chemotherapy, one was in stage II while the rest were in stage III. Therefore, we compared survival in subjects with stage III disease (n=51 and 68 for those with vs without neoadjuvant chemotherapy, respectively). No significant difference in OS (34.0 vs 33.0 months; P=0.33) and DFS (31.0 vs 29.0 months; P=0.27) was found between these two subgroups. Among the patients treated with adjuvant chemotherapy, only patients in stage III showed OS and DFS superior to that of patients without adjuvant chemotherapy (OS: 35.0 vs 25.0 months; P=0.026; DFS: 31.0 vs 23.0 months; P=0.036) (Figure 5). We next examined the influence of adjuvant chemotherapy according to *FGFR1* amplification status. Thirty-six patients with *FGFR1* amplification received adjuvant therapy. Compared to those with *FGFR1* amplification receiving no adjuvant chemotherapy, these patients had significantly longer median OS (chemotherapy: 42.0 vs no chemotherapy 26.0 months; *P*=0.006) and median DFS (chemotherapy 38.0 vs no chemotherapy: 21.0 months; P=0.009) (Figure 5). Two hundred sixty-six patients without *FGFR1* amplification received adjuvant therapy and showed no significant difference in OS (chemotherapy: 56.0 vs no chemotherapy 47.0 months; *P*=0.81) and DFS (chemotherapy: 53.0 vs no chemotherapy 42.0 months; *P*=0.84) compared to 223 patients without *FGFR1* amplification receiving no adjuvant chemotherapy. However, no significant difference was observed in OS and DFS between patients with or without adjuvant radiotherapy.

### Alcohol intake and *FGFR1* amplification

**Figure 6:**
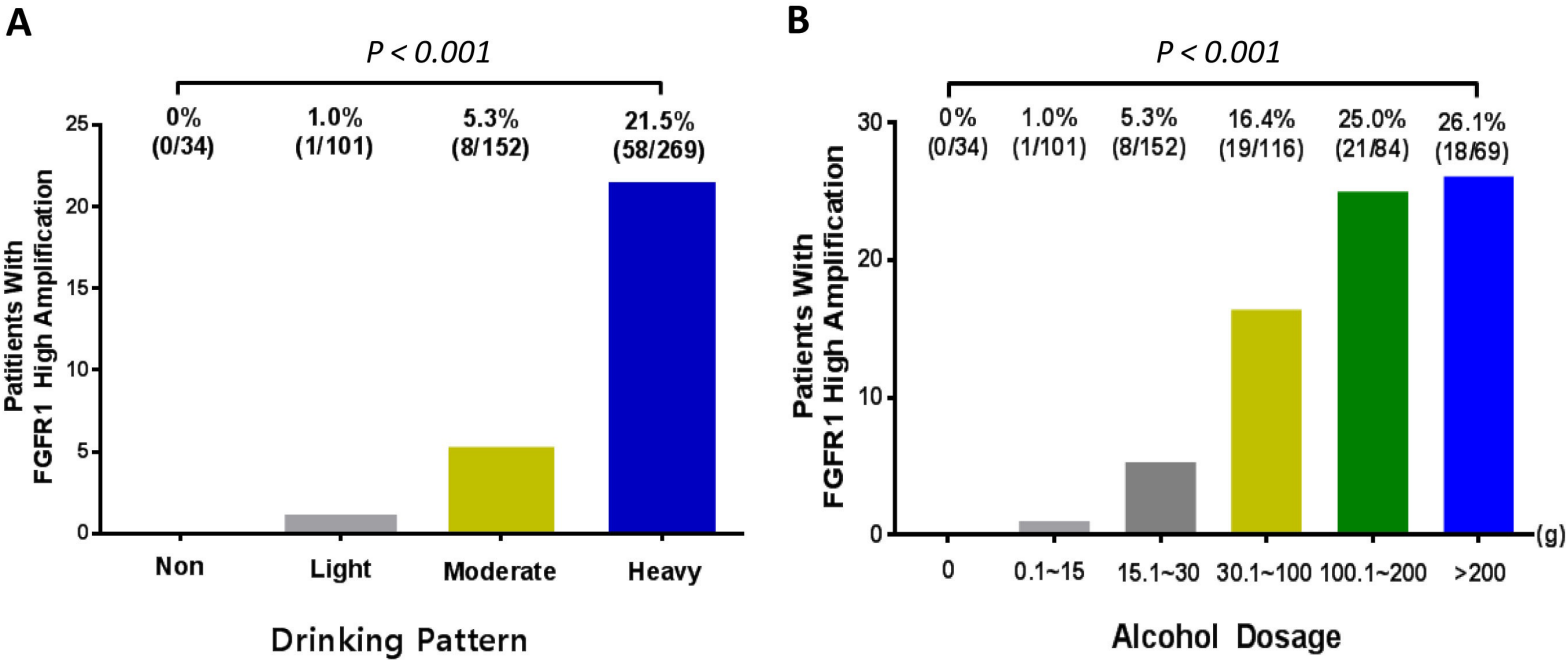
The incidence of FGFR1 amplification according to **(A)** drinking pattern and **(B)** alcohol dosage. P value was tested by χ2 test for linear trend.

Given the high percentage of heavy drinkers (48.4%) in the study sample, we examined the rate of *FGFR1* amplification according to alcohol intake. We found higher rate of *FGFR1* amplification in heavy drinkers (21.5%) than light (1.0%) or moderate drinkers (5.3%). Furthermore, the incidence of *FGFR1* amplification was 26.1% in patients whose alcohol intake was more than 200 g/day, 25.0% in patients whose alcohol intake was 100.1 to 200 g/day, 16.4% in patients whose alcohol intake was 30.1 to 100 g/day, 5.3% in patients whose alcohol intake was 15.1 to 30 g/day, and 1.0% in patients whose alcohol intake was less than 15 g/day, suggesting increasing rate of *FGFR1* amplification with increment of alcohol intake (*P*_*trend*_< 0.001) (Figure 6).

## DISCUSSION

FGFR1 is an important signaling molecule implicated in multiple cellular process including cellular growth and survival as well as angiogenesis. *FGFR1* amplification has been documented in ESCC and other cancer types; however, it still remains controversial whether *FGFR1* amplification adversely impacts on the clinical outcome of ESCC patients [13–15]. In this study, we investigated a large cohort of 556 ESCC patients who received radical resection of curative intent at our institution and whose *FGFR1* amplification was known by FISH. We demonstrated that *FGFR1* amplification was an independent adverse prognostic predictor of OS and DFS of ESCC patients. This finding was further supported by our immunohistochemical analysis showing that high intratumoral FGFR1 expression correlated with significantly shorter OS and DFS of ESCC patients.

*FGFR1* amplification status has been investigated in several previous studies using different approaches such as comparative genomic hybridization, single nucleotide polymorphism array, and FISH assay [20–23]. Unlike EGFR mutation in lung cancer, which is more prevalent in Asians than in Caucasians, the frequency of *FGFR1* amplification in ESCC does not seem to be widely different by ethnicity. The frequency of FGFR1 amplification, as detected by FISH analysis in our cohort, was 12.1%, which was higher than 9.4% reported by Loga *et al.* for a Caucasian ESCC patients cohort, [13] but lower than 21.4% for a small Korean cohort reported by Kwon *et al* [14].

In head and neck squamous cell carcinoma, *FGFR1* amplification was significantly associated with poor prognostic factors such as higher T stage, and visceral metastasis [24]. In lung squamous cell carcinoma, Kim *et al.* found that *FGFR1* amplification was an adverse prognostic factor while Tran *et al.* reported the opposite outcomes [25, 26]. Tumor heterogeneity, varied assaying methods, lack of consensus criteria for *FGFR1* amplification and small sample size may contribute to the controversial results. Current evidence on the prognostic significance of *FGFR1* amplification for ESCC also remains inclusive. Two studies of smaller cohort size yielded conflicting results. Loga *et al.* found no association between *FGFR1* amplification and clinical outcome of ESCC patients while Kwon *et al.* showed that *FGFR1* amplification was an independent predictor of prolonged OS of ESCC patients [13, 14]. Kim *et al.* investigated 526 curatively resected ESCC and showed that high *FGFR1* amplification was associated with significantly shorter DFS and OS [15]. Consistently, our study of 556 ESCC patients also showed that *FGFR1* amplification was independently associated with worse OS and DFS. On the basis of previous studies, we adopted a criteria including both of gene copy number and *FGFR1*/CEN8 ratio in our study [23]. High *FGFR1*/CEN8 ratio has been used to screen patients of squamous cell lung cancer who may benefit from treatment with FGFR inhibitors [27, 28]. Apart from genetic evidence, we further showed that high intratumoral FGFR1 expression was also associated with shorter OS and DFS, lending support to the proposition that *FGFR1* amplification predicts poor clinical outcome of ESCC patients.

The genomic landscape of ESCC is highly complex [29]. Delineation of aberrant signaling pathways in ESCC has identified candidate molecular targets such as HER2, EGFR, VEGF, FGFR and PI3K. However, lack of reliable prognostic predictors hampers development of targeted therapies for ESCC. Our study has demonstrated that *FGFR1* amplification is an independent adverse predictor of OS of ESCC patients, suggesting that FGFR1 may be a promising molecular therapeutic target [30]. Defining therapeutic targets for subgroups of ESCC patients is critical to advancing treatment of this disease. Currently, *FGFR1* amplification may define one large subgroup, and efforts to target this population are already ongoing with some clinical trials already in progress. FGFR1 inhibitors that are currently in clinical development are shown in Supplementary Table 2. Dovitinib, a potent FGFR inhibitor, has demonstrated antitumor activity in heavily pretreated patients with FGFR pathway-amplified breast cancer but has not been studied in clinical trials for ESCC patients [31].

Patients who would benefit from postoperative adjuvant chemotherapy in ESCC are still undefined [32–34]. Our data showed the benefit of adjuvant chemotherapy in patients with stage III ESCC. Patients with *FGFR1* amplification benefitted from adjuvant chemotherapy while patients without *FGFR1* amplification did not. However, this result should be cautiously interpreted because of the small sample size and, in addition, *FGFR1* amplification may be a local manifestation of global genomic instability. Concurrent genetic alterations in other regions of the genome associated with DNA damage/repair may contribute to greater benefit from adjuvant chemotherapy [35]. Therefore, comprehensive evaluation of genomic alterations will be needed to develop optimized treatment decisions for patients with resected ESCC.

Alcohol intake is an established risk for many types of cancer including ESCC while how it may affect the gene is still unknown [21, 36]. A novel finding of the current study is significant association between *FGFR1* amplification and alcohol intake. We also found that the incidence of F*GFR1* amplification rose as the amount of alcohol intake increased. Our findings suggest that *FGFR1* amplification may be a major oncogenic aberration in ESCC that is induced by alcohol abuse. Interestingly, among heavy drinkers with more than 100g per day, approximately 20% showed *FGFR1* amplification. On the basis of this finding, ESCC patients with alcohol consumption more than 100 g/day may be targeted for screening for *FGFR1* amplification.

The main limitations of this study reside in its retrospective nature and the fact that patients were selected from a single tertiary care institution. Also, clinical decisions were not likely uniform in such retrospective study, and could have produced biases. Future studies are required to validate our findings in an independent cohort, especially in a Caucasian population.

In this study, we demonstrated that *FGFR1* amplification is an independent adverse prognostic factor in surgically resected ESCC, indicating that *FGFR1* amplification may be a relevant therapeutic target in ESCC. *FGFR1* amplification was positively associated with alcohol consumption, which strongly implies that *FGFR1* amplification is an oncogenic aberration caused by alcohol abuse.

## MATERIALS AND METHODS

### Patients

We retrospectively analyzed the clinicopathologic data of patients with pathologically proven primary thoracic ESCC who received radical resection of curative intent at Shandong Provincial Hospital affiliated to Shandong University, Jinan, China, between January 2004 and December 2009. Patients with incomplete survival data were excluded. Archived tumor specimens were obtained from the Tissue Bank of Shandong Provincial Hospital. ESCC was staged according to the American Joint Committee on Cancer (seventh edition) guidelines.

The study protocol was approved by the Ethics Committee of Shandong Provincial Hospital. Patient consent was not required because of the retrospective nature of this study. Acquisition of tissue specimens was approved by the Shandong Provincial Hospital Ethics Committee and performed in accordance with the established institutional and state guidelines.

### Patient assessment

We retrieved data, including age, gender, co-morbidities, smoking and drinking status, TNM stage, OS and DFS, from the medical records of eligible patients. Never-smokers were defined as those smoking fewer than 100 cigarettes in lifetime; former smokers were those who had stopped smoking for more than one year, and current smokers were those who currently smoke or quit smoking for less than one year [37]. Alcohol intake was categorized into light drinker (0-15g/day), moderate drinker (15.1-30g/day) and heavy drinker (>30 g/day) [38]. Diabetes was defined as fasting blood glucose ≥ 7.0 mmol/L or 2-hour plasma glucose ≥ 11.1 mmol/L or previously diagnosed diabetes. Hypertension was defined as seated resting systolic blood pressure ≥ 140 mmHg or diastolic blood pressure ≥ 90 mmHg, or taking an anti-hypertensive medication [39].

### Follow-up assessment

All patients were followed up every 3 months for the first 3 years, every 6 months for the following 3 years, and thereafter annually. Follow-up was done at the Out-Patient Department of Shandong Provincial Hospital or by phone calls or postal mails if the patients failed to appear or missed appointments. Survival was measured from the date of surgery to the date of death or the last follow-up. The last follow-up visit was carried out in June 2015. DFS was defined as the length of time from the date of surgery to the date of initial tumor relapse (local/distant recurrence) or death as a result of any cause. OS was calculated from the time of surgery to death or the last follow-up date.

### Gene copy number analysis

Paraffin-embedded tumor specimens were used to construct a tissue microarray with 2-mm-diameter cores. Two pathologists (Y.J.Q. and Q.X.W.) confirmed the diagnosis of ESCC by hematoxylin and eosin (H&E) staining. FISH assay was performed on the tissue microarrays by using a ZytoLight SPEC *FGFR1*/CEN 8 Dual Color Probe (ZytoVision, Bremerhaven, Germany) according to the manufacturer’s recommended protocol. All samples were independently analyzed by two evaluators (Y.J.Q. and Q.X.W.) who were blinded to clinical data using a fluorescence microscope (DM4000, Leica). At least 100 nuclei per sample were evaluated. *FGFR1* amplification cases were defined as harboring a gene copy number ≥ 6 or *FGFR1*/CEN 8 ratio ≥ 2.2 [40]. Images were produced using the AxioCamMRm CCD camera and Axiovision v4.5 software suite.

### Immunohistochemistry

Immunohistochemical analysis was performed using anti-FGFR1 antibody (GTX100264, GeneTex, U.S.A.) following conventional methods. FGFR1 expression levels were scored semi-quantitatively independently by two pathologists (Y.J.Q. and Q.X.W.) who were blinded to patient information. Only clear membranous staining of tumor cells was considered to be positive; cytoplasmic or granular staining was considered negative or trace. The percentage of positive tumor cells per core (0% to 100%) was multiplied by the dominant staining pattern (1, negative or trace; 2, weak; 3, moderate; 4, intense). The overall score theoretically ranged from 0 to 400 [41]. The cutoff value for IHC scores was determined using the X-tile software (version 3.6.1) to stratify ESCC patients into the high and low FGFR1 expression groups [42]. Cases with discordant results underwent a consensus review by a third pathologist (Z.G.Y.).

### Statistical analysis

Patient demographic and baseline characteristics are expressed as mean (SD) or percentage (%) and compared using Student’s *t*-test and χ^2^ test, where appropriate. The primary end point of OS and secondary end point of DFS are presented as median and 95% confidence interval (95%CI), and analyzed using the Kaplan-Meier method, followed by log rank test. Statistical significance was set at *P<0.05*. All statistical analyses were performed by using SPSS 19.0 software.

## SUPPLEMENTARY MATERIALS FIGURES AND TABLES



## Abbreviations

FGFR1: Fibroblast growth factor receptor 1
ESCC: Esophageal squamous cell carcinoma
FISH: Fluorescent *in situ* hybridization
OS: Overall survival
DFS: Disease-free survival
AHR: Adjusted hazard ratio
EAC: Esophageal adenocarcinoma
IHC: Immunohistochemistry
PI3K: Phosphatidylinositol 3-kinase
AKT: Protein kinase B
MAPK: Mitogen-activated protein kinase

## References

[1] Ferlay J, Shin HR, Bray F, Forman D, Mathers C, Parkin DM (2010). Estimates of worldwide burden of cancer in 2008: GLOBOCAN 2008. Int J Cancer.

[2] Torre LA, Bray F, Siegel RL, Ferlay J, Lortet-Tieulent J, Jemal A (2015). Global cancer statistics, 2012. CA Cancer J Clin.

[3] Zhang Y (2013). Epidemiology of esophageal cancer. World J Gastroenterol.

[4] Shaheen N, Ransohoff DF (2002). Gastroesophageal reflux, barrett esophagus, and esophageal cancer: scientific review. JAMA.

[5] Holmes RS, Vaughan TL (2007). Epidemiology and pathogenesis of esophageal cancer. Semin Radiat Oncol.

[6] Belkhiri A, El-Rifai W (2015). Advances in targeted therapies and new promising targets in esophageal cancer. Oncotarget.

[7] Song Y, Li L, Ou Y, Gao Z, Li E, Li X, Zhang W, Wang J, Xu L, Zhou Y, Ma X, Liu L, Zhao Z (2014). Identification of genomic alterations in oesophageal squamous cell cancer. Nature.

[8] Zhang L, Zhou Y, Cheng C, Cui H, Cheng L, Kong P, Wang J, Li Y, Chen W, Song B, Wang F, Jia Z, Li L (2015). Genomic analyses reveal mutational signatures and frequently altered genes in esophageal squamous cell carcinoma. Am J Hum Genet.

[9] Freier K, Schwaenen C, Sticht C, Flechtenmacher C, Muhling J, Hofele C, Radlwimmer B, Lichter P, Joos S (2007). Recurrent FGFR1 amplification and high FGFR1 protein expression in oral squamous cell carcinoma (OSCC). Oral Oncol.

[10] Reis-Filho JS, Simpson PT, Turner NC, Lambros MB, Jones C, Mackay A, Grigoriadis A, Sarrio D, Savage K, Dexter T, Iravani M, Fenwick K, Weber B (2006). FGFR1 emerges as a potential therapeutic target for lobular breast carcinomas. Clin Cancer Res.

[11] Weiss J, Sos ML, Seidel D, Peifer M, Zander T, Heuckmann JM, Ullrich RT, Menon R, Maier S, Soltermann A, Moch H, Wagener P, Fischer F (2010). Frequent and focal FGFR1 amplification associates with therapeutically tractable FGFR1 dependency in squamous cell lung cancer. Sci Transl Med.

[12] Turner N, Grose R (2010). Fibroblast growth factor signalling: from development to cancer. Nat Rev Cancer.

[13] Loga KV, Kohlhaussen J, Marx AH, Sauter G, Grob T, Quaas A (2013). FGFR1 amplification is linked to the squamous cell carcinoma subtype in esophageal carcinoma. Cancer Res.

[14] Kwon D, Yun JY, Keam B, Kim YT, Jeon YK (2016). Prognostic implications of FGFR1 and MYC status in esophageal squamous cell carcinoma. World J Gastroenterol.

[15] Kim HS, Lee SE, Bae YS, Kim DJ, Lee CG, Hur J, Chung H, Park JC, Jung DH, Shin SK, Lee SK, Lee YC, Kim HR (2015). Fibroblast growth factor receptor 1 gene amplification is associated with poor survival in patients with resected esophageal squamous cell carcinoma. Oncotarget.

[16] Turner NC, Seckl MJ (2010). A therapeutic target for smoking-associated lung cancer. Sci Transl Med.

[17] Dutt A, Ramos AH, Hammerman PS, Mermel C, Cho J, Sharifnia T, Chande A, Tanaka KE, Stransky N, Greulich H, Gray NS, Meyerson M (2011). Inhibitor-sensitive FGFR1 amplification in human non-small cell lung cancer. PLoS One.

[18] Andre F, Bachelot T, Campone M, Dalenc F, Perez-Garcia JM, Hurvitz SA, Turner N, Rugo H, Smith JW, Deudon S, Shi M, Zhang Y, Kay A (2013). Targeting FGFR with dovitinib (TKI258): preclinical and clinical data in breast cancer. Clin Cancer Res.

[19] Tabernero J, Bahleda R, Dienstmann R, Infante JR, Mita A, Italiano A, Calvo E, Moreno V, Adamo B, Gazzah A, Zhong B, Platero SJ, Smit JW (2015). Phase I dose-escalation study of JNJ-42756493, an oral pan-fibroblast growth factor receptor inhibitor, in patients with advanced solid tumors. J Clin Oncol.

[20] Ishizuka T, Tanabe C, Sakamoto H, Aoyagi K, Maekawa M, Matsukura N, Tokunaga A, Tajiri T, Yoshida T, Terada M, Sasaki H (2002). Gene amplification profiling of esophageal squamous cell carcinomas by DNA array CGH. Biochem Biophys Res Commun.

[21] Weiss MM, Kuipers EJ, Hermsen MA, van Grieken NC, Offerhaus J, Baak JP, Meuwissen SG, Meijer GA (2003). Barrett's adenocarcinomas resemble adenocarcinomas of the gastric cardia in terms of chromosomal copy number changes, but relate to squamous cell carcinomas of the distal oesophagus with respect to the presence of high-level amplifications. J Pathol.

[22] Bandla S, Pennathur A, Luketich JD, Beer DG, Lin L, Bass AJ, Godfrey TE, Litle VR (2012). Comparative genomics of esophageal adenocarcinoma and squamous cell carcinoma. Ann Thorac Surg.

[23] Bass AJ, Watanabe H, Mermel CH, Yu S, Perner S, Verhaak RG, Kim SY, Wardwell L, Tamayo P, Gat-Viks I, Ramos AH, Woo MS, Weir BA (2009). SOX2 is an amplified lineage-survival oncogene in lung and esophageal squamous cell carcinomas. Nat Genet.

[24] Goke F, Bode M, Franzen A, Kirsten R, Goltz D, Goke A, Sharma R, Boehm D, Vogel W, Wagner P, Lengerke C, Kristiansen G, Kirfel J (2013). Fibroblast growth factor receptor 1 amplification is a common event in squamous cell carcinoma of the head and neck. Mod Pathol.

[25] Kim HR, Kim DJ, Kang DR, Lee JG, Lim SM, Lee CY, Rha SY, Bae MK, Lee YJ, Kim SH, Ha SJ, Soo RA, Chung KY (2013). Fibroblast growth factor receptor 1 gene amplification is associated with poor survival and cigarette smoking dosage in patients with resected squamous cell lung cancer. J Clin Oncol.

[26] Tran TN, Selinger CI, Kohonen-Corish MR, McCaughan BC, Kennedy CW, O'Toole SA, Cooper WA (2013). Fibroblast growth factor receptor 1 (FGFR1) copy number is an independent prognostic factor in non-small cell lung cancer. Lung Cancer.

[27] Sequist LV, Cassier P, Varga A, Tabernero J, Schellens JH, Delord JP (2014). Phase I study of BGJ398, a selective pan-FGFR inhibitor in genetically preselected advanced solid tumors. Cancer Res.

[28] Paik PK, Shen R, Ferry D, Soria JC, Mathewson A, Kilgour E, Rooney C, Smith NR, Cullberg M, Kilgour E, Landers D, Frewer P, Brooks N, André F (2014). A phase 1b open-label multicenter study of AZD4547 in patients with advanced squamous cell lung cancers: preliminary antitumor activity and pharmacodynamic data. J Clin Oncol.

[29] Sawada G, Niida A, Uchi R, Hirata H, Shimamura T, Suzuki Y, Shiraishi Y, Chiba K, Imoto S, Takahashi Y, Iwaya T, Sudo T, Hayashi T (2016). Genomic landscape of esophageal squamous cell carcinoma in a Japanese pGastroenterology.

[30] Giacomini A, Chiodelli P, Matarazzo S, Rusnati M, Presta M, Ronca R (2016). Blocking the FGF/FGFR system as a “two-compartment” antiangiogenic/antitumor approach in cancer therapy. Pharmacol Res.

[31] Musolino A, Campone M, Neven P, Denduluri N, Barrios CH, Cortes J, Blackwell K, Soliman H, Kahan Z, Bonnefoi H, Squires M, Zhang Y, Deudon S (2017). Phase II, randomized, placebo-controlled study of dovitinib in combination with fulvestrant in postmenopausal patients with HR+, HER2- breast cancer that had progressed during or after prior endocrine therapy. Breast Cancer Res.

[32] Gao SJ, Park HS, Corso CD, Rutter CE, Kim AW, Johung KL (2017). Role of adjuvant treatment esophageal cancer with incidental pathologic node positivity. Ann Thorc Surg.

[33] Zhang L, Li W, Lyu X, Song Y, Mao Y, Wang S, Huang J (2017). Adjuvant chemotherapy with paclitaxel and cisplatin in lymph node-positive thoracic esophageal squamous cell carcinoma. Chin J Cancer Res.

[34] Klevebro F, Ekman S, Nilsson M (2017). Current trends in multimodality treatment of esophageal and gastroesophageal junction cancer - review article. Surg Oncol.

[35] Zhang W, Mao JH, Zhu W, Jain AK, Liu K, Brown JB, Karpen GH (2016). Centromere and kinetochore gene misexpression predicts cancer patient survival and response to radiotherapy and chemotherapy. Nat Commun.

[36] Urashima M, Hama T, Suda T, Suzuki Y, Ikegami M, Sakanashi C, Akutsu T, Amagaya S, Horiuchi K, Imai Y, Mezawa H, Noya M, Nakashima A (2013). Distinct effects of alcohol consumption and smoking on genetic alterations in head and neck carcinoma. PLoS One.

[37] Couraud S, Zalcman G, Milleron B, Morin F, Souquet PJ (2012). Lung cancer in never smokers--a review. Eur J Cancer.

[38] Cao Y, Willett WC, Rimm EB, Stampfer MJ, Giovannucci EL (2015). Light to moderate intake of alcohol, drinking patterns, and risk of cancer: results from two prospective US cohort studies. BMJ.

[39] Peng F, Hu D, Lin X, Chen G, Liang B, Zhang H, Dong X, Lin J, Zheng X, Niu W (2017). Analysis of preoperative metabolic risk factors affecting the prognosis of patients with esophageal squamous cell carcinoma: The Fujian Prospective Investigation of Cancer (FIESTA) Study. EBioMedicine.

[40] Wolff AC, Hammond ME, Schwartz JN, Hagerty KL, Allred DC, Cote RJ, Dowsett M, Fitzgibbons PL, Hanna WM, Langer A, McShane LM, Paik S, Pegram MD (2007). American Society of Clinical Oncology/College of American Pathologists guideline recommendations for human epidermal growth factor receptor 2 testing in breast cancer. J Clin Oncol.

[41] Hirsch FR, Varella-Garcia M, Bunn PA, Di Maria MV, Veve R, Bremmes RM, Barón AE, Zeng C, Franklin WA (2003). Epidermal growth factor receptor in non-small-cell lung carcinomas: correlation between gene copy number and protein expression and impact on prognosis. J Clin Oncol.

[42] Camp RL, Dolled-Filhart M, Rimm DL (2004). X-tile: a new bio-informatics tool for biomarker assessment and outcome-based cut-point optimization. Clin Cancer Res.

