# Supplementary material for: Association of fibroblast growth factor receptor 1 gene amplification with poor survival in patients with esophageal squamous cell carcinoma
Source: Oncotarget. 2017 Oct 4;8(51):88857–69. doi: 10.18632/oncotarget.21486 (PMC5687652; doi:10.18632/oncotarget.21486)
Supplement: Supplementary file 1 [file oncotarget-08-88857-s001.pdf]

Association of fibroblast growth factor receptor 1 gene amplification with poor survival in patients with esophageal squamous cell carcinoma

SUPPLEMENTARY MATERIALS

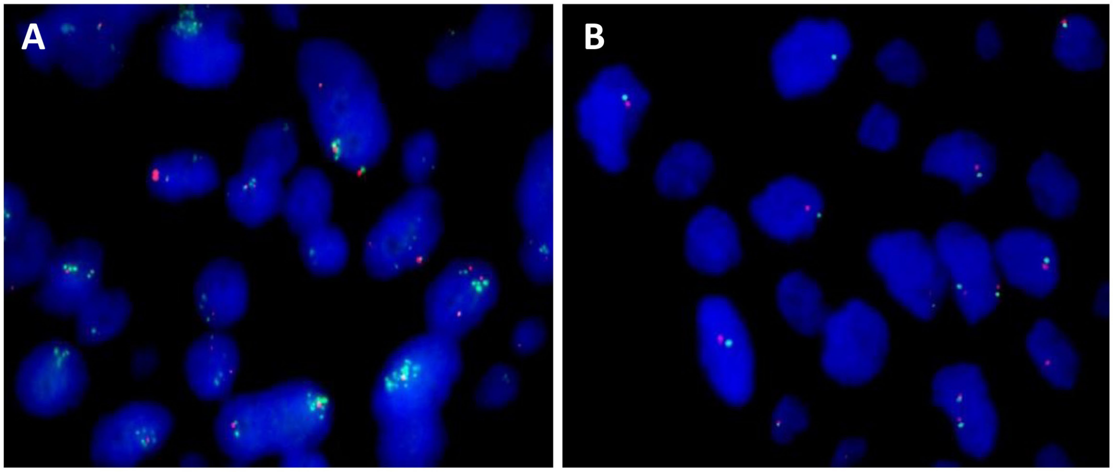

Supplementary Figure 1: Fibroblast growth factor receptor 1 (FGFR1) amplification assessed by fluorescent *in situ* hybridization (FISH). **(A)** *FGFR1* amplification (+); **(B)** *FGFR1* amplification (-).

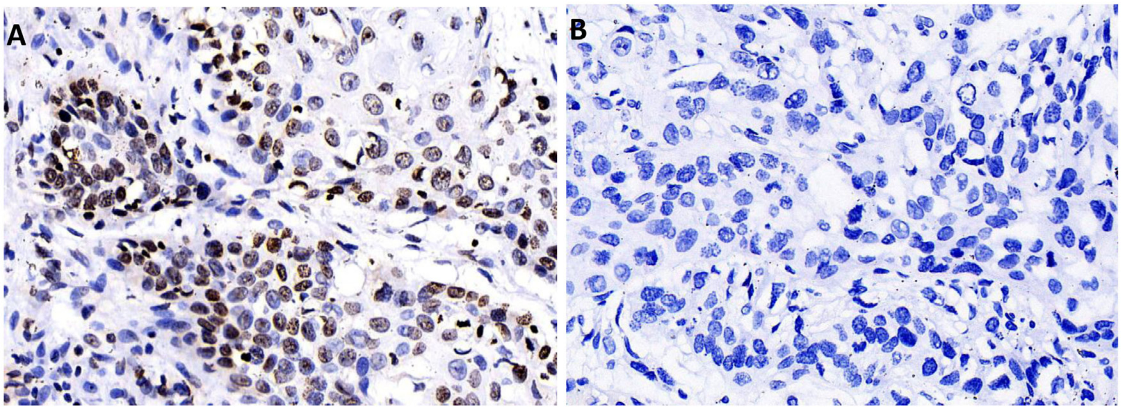

Supplementary Figure 2: P53 expression in esophageal squamous cell carcinomas assessed by immunohistochemistry (magnification: ×200). **(A)** p53 positive. **(B)** p53 negative.

Supplementary Table 1: Patient demographic and baseline characteristics stratified by high and low FGFR1 expression by immunohistochemistry

Characteristics	FGFR1 expression		*P*
	High	Low	
**No. of patients (%)**	81(14.6)	475(85.4)	
**Median age (range), years**	63(45-72)	63(39-80)	0.48
**Male sex, n(%)**	74(91.4)	405(85.3)	0.14
**Tumor location, n(%)**			0.37
Upper	0(0.0)	9(21.9)	
Middle	67(83.6)	362(76.3)	
Lower	14(16.4)	104(21.9)	
**Median tumor size (range), cm**	3.5(1.0-8.0)	3.0(0.5-9.0)	0.82
**pT stage, n(%)**			0.10
T1	19(23.4)	100(21.1)	
T2	30(37.0)	235(49.5)	
T3	24(29.6)	117(24.6)	
T4	8(9.9)	23(4.8)	
**pN stage, n(%)**			0.46
N0	27(33.3)	183(38.5)	
N1	39(48.1)	232(48.8)	
N2	10(12.3)	36(7.6)	
N3	5(6.2)	24(5.1)	
**pTNM stage, n(%)**			0.16
I	22(27.1)	91(19.2)	
II	40(49.4)	284(59.8)	
III	19(23.5)	100(21.0)	
**Differentiation, n(%)**			0.14
Well	27(33.3)	150(31.6)	
Moderate	35(43.2)	251(52.8)	
Poorly	19(23.5)	74(15.6)	
**P53 expression, n(%)**			0.32
Positive	38(46.9)	251(52.8)	
**Diabetes, n(%)**			0.33
Yes	14(13.4)	63(13.9)	
**Hypertension, n(%)**			0.76
Yes	29(35.8)	162(34.1)	
**Smoking status, n(%)**			0.10
Never-smoker	12(14.8)	112(23.5)	
Former smoker	22(27.2)	91(19.2)	
Current smoker	47(58.0)	272(57.3)	
**Alcohol intake, n(%)**			0.00
None	0(0)	34(7.2)	
Light	2(2.5)	99(20.8)	
Moderate	15(18.5)	137(28.8)	
Heavy	64(79.0)	205(43.2)	

Supplementary Table 2: FGFR1 inhibitors currently in clinical trials

Drug	Company	IC_{50}	Phase	Other targets	Ref
AZD4547	AstraZeneca	0.2nM	II	FGFR2, FGFR3, FGFR4, KDR, IGFR	[43]
BGJ398	Novartis	0.9nM	II	FGFR2, FGFR3, FGFR4, VEGFR2, Lyn, Kit	[44]
Erdafitinib	Medchemexpress	<1nM	II	FGFR2, FGFR3, FGFR4	[45]
Ponatinib	Ariad	2.2nM	II	FGFR2, FGFR3, FGFR4, Abl, PDGFRα, VEGFR2, c-Src	[46]
TAS-120	Taiho Oncology	3.9nM	II	FGFR2, FGFR3, FGFR4	[47]
Dovitinib	Novartis	8.0nM	II	FGFR2, FGFR3, FGFR4, FLT3, c-Kit, VEGFR3	[48]
ASP5878	AstellasPharma	0.5nM	I	FGFR2, FGFR3, FGFR4	[49]
E7090	Eisai	0.7nM	I	FGFR2, FGFR3, FGFR4	[50]
LY2874455	Lilly	2.8nM	I	FGFR2, FGFR3, FGFR4, VEGFR2	[51]
Debio1347	Debiopharm	9.3nM	I	FGFR2, FGFR3, FGFR4	[52]
